# Prevalence and novel risk factors for vitamin D insufficiency in elite athletes: systematic review and meta-analysis

**DOI:** 10.1007/s00394-022-02967-z

**Published:** 2022-07-26

**Authors:** Tilda Harju, Blair Gray, Alexandra Mavroedi, Abdulaziz Farooq, John Joseph Reilly

**Affiliations:** 1 University of Strathclyde School of Psychological Sciences & Health, Glasgow, Scotland; 2 FIFA Medical Centre of Excellence, Aspetar, Orthopaedic and Sports Medicine Hospital, Doha, Qatar

**Keywords:** Sport, Nutrition, Vitamin D, Nutritional status

## Abstract

**Background and purpose:**

Vitamin D insufficiency may be common among elite athletes, but prevalence is unclear, and some potentially important risk factors are uncertain. The present study aimed to (a) estimate the prevalence of vitamin D insufficiency in elite athletes, and (b) examine differences in prevalence between the sexes, and between adults and adolescents, from recent studies which used a contemporary definition of insufficiency.

**Methods:**

Four databases (Web of Science, SPORTDiscus, PubMed, and Sports Medicine and Education Index) were searched for studies in elite athletes. Literature selection, data extraction, and risk of bias assessment were conducted independently by two researchers. Vitamin D insufficiency was defined as 25(OH)D < 50 nmol/L. Meta-analysis was conducted, using R software x64 4.0.2, to provide estimates of prevalence of insufficiency for adults and adolescents, and to examine between-sex differences in risk of insufficiency.

**Results:**

From the initial 943 literature search hits, 51 studies were eligible with 5456 participants, 33 studies in adults (12/33 in winter and spring), 15 studies in adolescents (6/15 in winter and spring) and 3 studies with age of study participants not given. Prevalence of vitamin D insufficiency from meta-analysis was 30% (95% CI 22–39%) in adults and prevalence was higher, though not significantly so, at 39% (95% CI 25–55%) in adolescents. Differences in the prevalence of insufficiency between the sexes for the eight studies which provided within-study comparisons was not significant (RR = 1.0; 95% CI 0.79–1.26). Evidence quality was moderate.

**Conclusions:**

Prevalence of vitamin D insufficiency (≤ 50 nmol/L) in elite athletes is high, suggesting a need for greater attention to prevention and treatment. Prevalence estimates in the present study are conservative due to a relative lack of studies in winter. While there was no evidence of higher risk among women than men in the present study, there was less evidence on women.

**Supplementary Information:**

The online version contains supplementary material available at 10.1007/s00394-022-02967-z.

## Introduction

Historically, vitamin D has been mainly recognised for its important role in calcium homeostasis and bone health. However, since the finding of vitamin D receptors in various different tissues, appreciation of other important functions of vitamin D has increased [1]. Today it is understood that vitamin D has a role in various non-skeletal functions, such as inflammation, the cardiovascular system, immune functions, and skeletal muscle function [2]. Vitamin D status is a hot topic in sports science [2]—it may influence musculoskeletal function, fracture risk and the recovery period from fractures, force and power production, and immune function [3]. All these factors are crucial for elite athletes as they may influence athletic performance, health in the short-term and long-term, and the ability to train [2, 3].

In the general population, the prevalence of vitamin D deficiency is high (from substantial minorities of the population to majorities in some studies), but variable between studies due to differences in the definition of deficiency, other methodological differences such as the assays used, and real differences between populations in latitude, season, and health behaviours related to sun exposure and dietary intake of vitamin D [2]. In elite sport, the prevalence of deficiency has been reported to be similarly high and variable, with prevalence estimates reaching 70–90% in some individual studies [4]. While there is widespread acceptance that vitamin D status can be assessed adequately by measuring serum 25-hydroxyvitamin D concentration [25(OH)D] [5, 6], the precise concentration of circulating 25(OH)D which indicates insufficiency or deficiency remain under debate. In recent years there has been greater consistency around a cut-off of 50 nmol/L to define insufficiency [7–9]. A recent review by the US Preventive Services Task Force [10] concluded that methodological differences between laboratories in the measurement of 25(OH)D concentrations is a barrier to assessment of vitamin D status.

Farrokhyar et al. reviewed studies on the prevalence of vitamin D insufficiency in sport participants, searching the literature to January 2014 [11] and identifying 23 eligible studies. To estimate the prevalence of vitamin D insufficiency in their eligible studies, Farrokhyar applied a definition of insufficiency of < 80 nmol/L 25OH(D). In the pooled sample, the prevalence above this cut-off was over 50%, and this review confirmed that two key risk factors for vitamin D insufficiency known from the general population (winter, living at high latitude) applied to elite sport [11], with relative risks of 1.85 for both participants measured during winter/spring relative to summer and autumn, and for participants at latitudes ≥ 40 °N vs those < 40 °N, though with substantial heterogeneity between studies which related at least partly to variations in sun exposure (e.g., whether participants trained and competed indoors or outdoors). Farrokhyar et al. [11] used a cut-off for 25(OH)D concentration (80 nmol/L) to define insufficiency which would be considered rather high today [7–9]. Since the completion of the literature search by Farrokhyar et al. in the beginning of 2014, the evidence based on the prevalence of vitamin D status in elite athletes has expanded substantially. There is increasing concern over possible risk factors for vitamin D insufficiency not addressed by Farrokhyar et al. [11], specifically differences in vitamin D status between males and females, and between adolescents and adults. While there is no clear evidence that elite sport increases risk of vitamin D insufficiency, recent increases in awareness of the issue in the general population and in sport, combined with greater policy emphasis on vitamin D supplementation and fortification, suggest that a focus on more recent studies will be necessary to understand current prevalence of insufficiency in specific population groups like participants in elite sport.

Females in sports science and sports medicine research have been under-represented as study participants in general [12]. Variation between males and females in vitamin D status might result from differences in body fatness, differences in sunlight exposure or other behavioural factors (athletic attire worn, sunscreen use, time of the training, and cloud cover) or differences in dietary intake, with consistently lower intake of vitamin D in females in the general population [3, 13]. While sun is the main source of vitamin D [2], vitamin D status is influenced meaningfully by dietary intake, particularly intake of supplements and vitamin D fortified foods. For example, in the UK women in the general population have significantly lower intakes of vitamin D fortified foods, and lower vitamin D status than men [14]; UK adolescents in the general population have lower intakes of vitamin D fortified foods than adults [14], and there is evidence that consumption of vitamin D supplements by adolescents in elite sport is negligible [15]. There is also some evidence that adolescent elite athletes perceive themselves to be at very low risk of vitamin D deficiency [16] which may influence their behaviour in relation to sun exposure and/or vitamin D intake. Adolescents in the general population may actually be at higher risk of insufficiency than adults [17, 18]*,* and may be more likely to benefit from vitamin D supplementation than adults [17].

The most recent definition of vitamin D insufficiency, rapid recent expansion of the evidence base, and emerging evidence of new potential risk factors for insufficiency, all suggest that an updated systematic review is needed to synthesize the evidence on prevalence, and to consider potential new risk factors. Therefore, the present study aimed to systematically review and appraise evidence on vitamin D insufficiency in elite athletic populations from 2014 to 2020, and to consider whether there are differences between males and females and between adolescents and adults.

## Methods

### Study design

The present systematic review is based on a predefined protocol and followed guidance from a 24-step checklist by Muka et al. [19]. The conduct and reporting also followed the PRISMA statement and checklist for systematic reviews [20] (PRISMA Checklist provided in Appendix 1). The protocol was registered in PROSPERO, registration code: CRD42020217898.

### Search strategy

The literature was searched using four relevant electronic databases: Web of Science, SPORTDiscus, PubMed, and Sports Medicine and Education Index. These specific databases were selected as the most appropriate for the search. The search terms followed the PECO (population, exposure, comparator, and outcome) outline [21] (Appendix 2). For each key term, various synonyms were utilized to allow a broader search, and the search terms from the previous systematic review [11] were taken into account. The search terms are presented in Table 1, and the same search strategy was used in all the four databases. The search was limited to only include studies published after January 2014 as the previous systematic review by Farrokhyar [11] included studies until then. The initial search was conducted in November 2020 and forward and backward citation searches in July 2021.

**Table 1:** Search terms used for all four databases

Row 1	Vitamin D OR Vitd OR 25-hydroxyvitamin D
Row 2	Elite OR professional
Row 3	Athlete* OR sport*
Row 4	Status OR level OR profile OR deficiency OR insufficiency OR hypovitaminosis D OR inadequacy

OR command utilized for items belonging to the same domain

*At the end of a word includes all words with the specific beginning to allow a broader search

### Study inclusion and exclusion criteria

The study population was elite athletes, but currently, there is no consensus definition of ‘elite athlete’ [22]. Therefore, for the purpose of this study, and to be as inclusive as possible, we accepted original study descriptions of ‘elite’; where no such description was given we defined ‘elite athlete’ as someone who represents a professional sports club or body and competes at either national or international level or is on a development pathway to such clubs and bodies, following the definition of an ‘elite athlete’ from the UK Government guidance [23]. Both male and female athletes were included, but athletes with serious ill health and/or disabilities were excluded to reduce the potential for bias in the study results (vitamin D status might have been affected by illness or disability). Other inclusion criteria were: serum 25(OH)D concentrations measured via blood draws, study published in English (due to resource limitations), peer-reviewed and full text available. The full eligibility criteria are provided in Appendix 3.

### Screening for eligibility

After completing the literature search on the four databases, references were imported to EndNote and duplicates removed. The inclusion criteria were then applied to the titles and abstracts, and full texts of the studies that met the eligibility criteria based on their titles and abstracts were retrieved. The full texts of potentially eligible studies were then screened by applying the selection criteria. The whole screening process was conducted by two researchers as a quality control measure to reduce errors in the screening process, with a third researcher utilized to resolve differences and discuss where needed.

### Data extraction

The data extraction form was based in part on the data tables in the previous systematic review [11]. The extracted data included the population demographics, sport, the season of the measurement, location, measurement method, vitamin D cut-offs used to define insufficiency, results for vitamin D status and serum 25(OH)D mean (SD), and the risk of bias score (Appendix 4.) Many variables relevant to vitamin D status were either not measured/collected, or not reported in most individual studies (e.g., sun exposure related variables such as indoor or outdoor training or competition; maturation status of the adolescent participants) and so could not be considered in the present study. The data were extracted from the eligible studies by the two researchers independently and cross-checked by a third.

If pooled means and standard deviations needed to be combined from different groups or studies, these were calculated with an online calculation tool (https://www.statstodo.com/index.php). From intervention studies that investigated the effect of supplementation, only the results of the baseline (pre-intervention) measures were extracted. If the study had two sample points and reported separate results, the prevalence for vitamin D insufficiency was calculated as an average of the number of athletes that had insufficient levels at any point. We used the WHO definition of adolescent (10.0–19.9 years inclusive) [24] and took the mean age in each study to classify participants as adolescents or adults where necessary. If age was not mentioned (and could not be obtained from the study corresponding author) then the study was included in the overall estimate of prevalence of insufficiency, but not included in the comparison of insufficiency prevalence between adults and adolescents.

There are differences in serum concentrations of 25(OH)D both between and within measurement methods [10, 25]. In the present study, the method of measurement was noted as ‘mass spectrometry’ or ‘immunoassay’ for simplicity, even if a specific type of mass spectrometry or immunoassay had been used. If the latitude was not reported in the article, an online platform (https://www.mapsofworld.com) was utilized to retrieve the approximate latitude of the country. If there was other information missing, the study authors were contacted to provide the necessary information. The extracted data were used for the descriptive synthesis of the results.

### Cut-off to define vitamin D insufficiency

Serum 25(OH)D concentrations can be reported in nanograms per milliliter (ng/mL) or nanomoles per liter (nmol/L) [8]. Most of the eligible studies reported the 25(OH)D concentrations in ng/mL or both ng/L and nmol/L. When the data were extracted from the eligible studies, all the results were converted to nmol/L utilizing a conversion formula: 1 ng/mL = 2.5 nmol/L [8]. The terminology and vitamin D cut-offs were extracted as they were defined in the original papers. The definitions and terminology relating to vitamin D deficiency and insufficiency have been unclear for some time [6]. Serum 25(OH)D level ≤ 50 nmol/L was chosen as the cut-off to define insufficiency for the present study as it was the lowest cut-off utilized in most eligible studies, there is a consensus that it indicates concerning vitamin D status [26], and has been recommended for use by US Endocrine Society [27] and the Institute of Medicine [7].

### Risk of bias assessment

The risk of bias assessment (methodological quality of the eligible studies) used the Joanna Briggs Institute Quality Appraisal Checklist for Prevalence Studies [28] (Appendix 5). This method was chosen because it is considered the most appropriate tool for assessing the methodological quality of prevalence studies [29]. The quality appraisal includes nine items (See Appendix 5), and the quality of each item is rated with ‘Yes’, ‘No’, ‘Unclear’, depending on how the component is completed. The overall quality score is the number of ‘Yes’ answers for the items (max score 9/9). Two researchers conducted risk of bias assessment independently and this was crossed checked against a third researcher in a subsample of 27% of the eligible studies and where the two researchers had doubts or differences.

### Statistical analysis

To estimate the prevalence of vitamin D insufficiency, a meta-analysis was performed, using method of assessment (Mass spectrometry and Immunoassay) as subgroups initially. A sensitivity analysis was conducted to exclude the studies that had a sample size less than 50 and to compare the results when all studies were considered. The level of heterogeneity in prevalence reported from studies was determined using the *I*^2^ statistic [30].

To compare the between-sex differences in vitamin D insufficiency within studies (to control for between-study differences in methodology for assessment of vitamin D status) only those studies that reported the prevalence separately for males and females, or with authors who provided this information when asked, were included (*n* = 8 studies, 5 in adults and 3 in adolescents). Meta-analysis was performed including these studies by reporting the risk ratio (RR) and 95% confidence intervals (CI). The presence of publication bias was investigated only for the meta-analysis comparing vitamin D insufficiency in males and females by plotting the funnel plots that shows the effect sizes on one axis against the observed variability on other axis. An *I*^2^ of 0–40% was considered low heterogeneity and fixed effects from meta-analysis were reported; when the *I*^2^ statistic was 75–100%. Heterogeneity was considerable, and a random effects model was used to report effect sizes. For continuous variables such as age and 25(OH)D concentration weighted means were computed based on the sample size of each study. All statistical analysis was conducted using the meta-library in the R software x64 4.0.2 (R Core Team (2020)).

## Results

### Literature searching and study selection

**Fig. 1:**
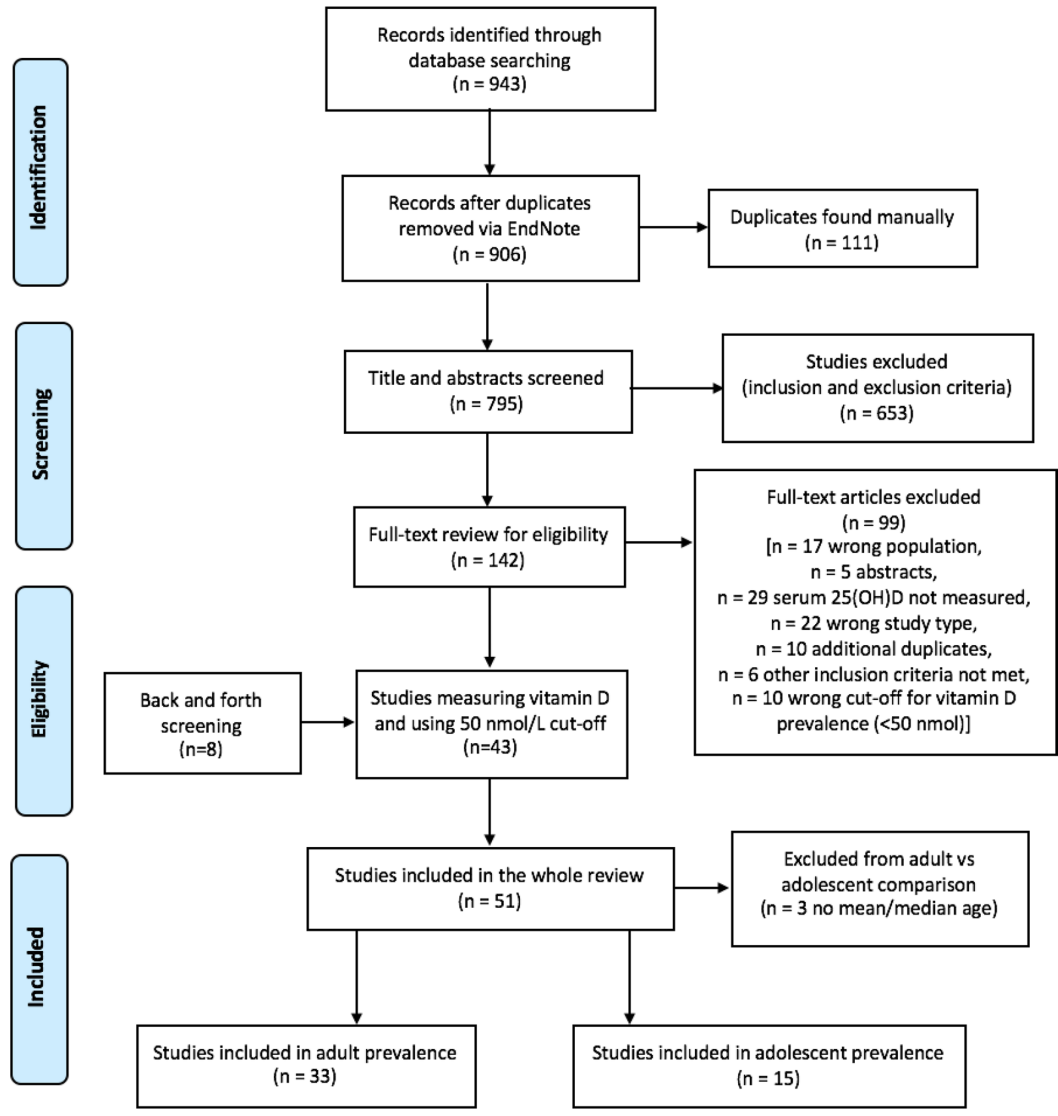
PRISMA flow diagram of the literature search and screening process

The PRISMA flow diagram presents the results from the literature search and the review process (Fig. 1). The initial search from the four databases yielded 943 studies. After applying the eligibility criteria to the titles and abstracts, 141 articles were eligible for full-text screening. Of these, 43 studies were eligible, and a further 8 eligible studies were added via forward and backward citation searching. The literature searching and screening process, therefore, resulted in a total of 51 eligible studies. To be able to conduct the adult vs adolescent comparison, studies were divided into two groups according the mean age: 33 studies had a mean age > 19 years old and 15 studies had a mean age < 19; 3 studies did not have mean age and, therefore, were excluded from the adult versus adolescent comparison.

### Study and sample characteristics—adult studies

Due to the large number of eligible studies, a summary table of the study characteristics and the quality assessment score for all the eligible adult studies is provided in Table 2. More detail with data extracted from each eligible study is presented in Appendix 6. The 33 eligible adult studies were published between January 2014 and September 2020 in 14 different countries. Immunoassay was the most common method utilized for measuring the serum 25(OH)D concentrations—26 studies used different kinds of immunoassays, and only 4 studies used mass spectrometry. Three studies did not report the method of measurement.

**Table 2:** Summary of eligible adult studies

Studies (*n*)	Participants (*n*)	Age [mean (SD)] gender [*n* (%)]	Type of sport (*n* of studies)	Location (*n* of studies)	Season of measurement (*n* of studies)	Serum 25(OH)D measurement method	Prevalence of vitamin D insufficiency [*n* (%)]	Weighted 25(OH)D level (nmol/L) [mean (SD)]	Quality score (mean)
33	3592	23.3 (4.9) M = 2713 (75.5) F = 754 (21.0) NG = 125 (3.5)	Indoor (17) Outdoor (10) Mixture (6)	Poland (8) US/Canada (6) Italy (4) South Korea, Uzbekistan, New Zealand, UK/Ireland (2) Netherlands, Qatar, Iran, Spain, Portugal, Turkey, Germany (1)	Summer (4) Winter (9) Spring (3) Fall (1) Multiple (13) NG (3)	IA (26) MA (4) NG (3)	1167 (32.5)	66.4 (30.4)	5/9

*M* males, *F* females, *NG* not given, *IA* immunoassay, *MA* mass spectrometry

The evidence base came from measurements across the year, 17 studies had a single sample point and 13 studies had two or more samples taken throughout the year. Three studies did not report the season of measurement. Overall, 12 out of the 30 eligible studies that recorded the season were conducted only during winter or spring, when prevalence of vitamin D insufficiency would have been highest.

The 33 eligible studies included 3592 athletes. The weighted mean (SD) age was 23.3 (4.9) years; from 3471 athletes from 30 studies that gave mean and standard deviation for age. Of all the athletes included in the adult studies, 76% were males, 21% were females, and for 3% of the athletes the sex/gender was not reported.

### Study and sample characteristics—adolescent studies

A summary table for adolescent studies is provided in Table 3. The 15 eligible adolescent studies are described in more detail in Appendix 7. The 15 eligible studies were published between February 2014 and February 2020 in 12 different countries*.* Immunoassay was the most common method utilized for measuring the serum 25(OH)D concentrations as ten studies used different kinds of immunoassays, three studies used mass spectrometry, and two studies did not report the method of measurement. The measurements were conducted during different seasons, nine studies had a single sample point and six studies had two or more samples taken throughout the year. Of the 15 studies, 6 took place in winter and/or spring only. The 15 eligible studies included 1432 athletes. The weighted mean (SD) age was 16.4 (2.6) (from 14 studies and 877 athletes). Of all adolescent athletes included, 56% were male and 41% were females, and for 3% of the athletes gender was not given.

**Table 3:** Summary of eligible adolescent studies

Studies (*n*)	Participants (*n*)	Age [mean (SD)] gender [*n* (%)]	Type of sport (*n* of studies)	Location (*n* of studies)	Season of measurement (*n* of studies)	Serum 25(OH)D measurement method	Prevalence of vitamin D insufficiency [*n* (%)]	Weighted 25 (OH) D level (nmol/L) [mean (SD)]	Quality score (mean)
15	1432	16.4 (2.6) M = 805 (56.2) F = 591 (41.3) NG = 36 (2.5)	Indoor (7) Outdoor (5) Mixture (3)	Poland (3) Germany (2) Turkey, Russia Brazil, Denmark, R.O. Korea, Sweden, Tunisia England, Israel, USA (1)	Summer (0) Winter (5) Spring (1) Fall (3) Multiple (6)	IA (10) MA (3) NG (2)	652 (45.5)	60.0 (33.6)	4/9

*M* males, *F* females, *NG* not given, *IA* immunoassay, *MA* mass spectrometry

### Risk of bias assessment

Table 4 presents the methodological quality assessments of the adult and adolescent studies. For the eligible adult studies (Table 4A), study quality score ranged from 2/9 to 8/9 with a mean of 5/9. Table 4B presents the quality assessment for adolescent studies—this ranged from 3/9 to 6/9 with a mean of 4/9.

**Table 4:** A Quality assessment of the eligible adult studies; B Quality assessment of the eligible adolescent studies

A
Critical appraisal items
1. Was the sample frame appropriate to address the target population?
2. Were study participants sampled in an appropriate way?
3. Was the sample size adequate?
4. Were the study subjects and the setting described in detail?
5. Was the data analysis conducted with sufficient coverage of the identified sample?
6. Were valid methods used for the identification of the condition?
7. Was the condition measured in a standard, reliable way for all participants?
8. Was there appropriate statistical analysis?
9. Was the response rate adequate, and if not, was the low response rate managed appropriately?

Study	1	2	3	4	5	6	7	8	9	Overall score
Allison et al. [31]	Yes	Unclear	Yes	Yes	Unclear	Yes	Yes	Yes	Yes	7/9
Backx et al. [32]	Yes	No	Yes	Yes	Unclear	Yes	Yes	Yes	Yes	7/9
Barcal et al. [33]	No	No	Yes	Yes	Yes	Yes	Yes	Yes	Yes	7/9
Bauer et al. [34]	Unclear	Unclear	No	Yes	Unclear	Yes	Yes	Yes	Unclear	4/9
Caroli et al. [35]	Unclear	Unclear	No	Yes	Unclear	Yes	Yes	Yes	Unclear	4/9
Fairbairn et al. [36]	No	Yes	Yes	Yes	Yes	Yes	Yes	Yes	Yes	8/9
Fileppella et al. [37]	Yes	Unclear	No	No	Unclear	Yes	Yes	Yes	Unclear	4/9
Fishman et al. [38]	Yes	Unclear	No	No	Unclear	Unclear	Unclear	Yes	Unclear	2/9
Hildebrand et al. [39]	No	No	Yes	Yes	Unclear	Yes	Yes	Yes	Yes	6/9
Jastrzebski et al. [40]	Unclear	Unclear	No	No	Unclear	Yes	Yes	Yes	Yes	4/9
Kerimov et al. [41]	Unclear	Unclear	No	No	Yes	Yes	Yes	Yes	Yes	5/9
Kim et al. [42]	No	Unclear	No	No	Unclear	Yes	Yes	Yes	Unclear	3/9
Kim et al. [43]	No	No	No	No	Unclear	Yes	Yes	Yes	Unclear	3/9
Kryzwanski et al. [44]	Yes	Unclear	No	Yes	Unclear	Yes	Yes	Yes	Yes	6/9
Ksiazek et al. [45]	No	Unclear	No	No	Unclear	Yes	Yes	Yes	Unclear	3/9
Ksiazek et al. [46]	No	Unclear	No	No	Unclear	Yes	Yes	Unclear	Unclear	2/9
Ksiazek et al. [47]	Unclear	Unclear	No	No	Unclear	Yes	Yes	Yes	Unclear	3/9
Lombardi et al. [48]	Unclear	Unclear	No	No	Unclear	Yes	Yes	Yes	Unclear	3/9
Malczewska. et al. [49]	Yes	Unclear	No	Yes	Yes	Yes	Yes	Yes	Yes	7/9
Maroon et al. [50]	No	No	No	Yes	Unclear	Unclear	Unclear	Yes	Yes	3/9
Mehran et al. [51]	No	Unclear	No	Yes	Unclear	Yes	Yes	Yes	Unclear	4/9
Parsaie et al. [52]	No	No	No	No	Unclear	Yes	Yes	Yes	Yes	4/9
Pietraszewska et al. [53]	Unclear	Unclear	No	No	Unclear	Yes	Yes	Yes	Unclear	3/9
Rebolledo et al. [54]	Yes	Yes	No	Yes	Unclear	Unclear	Unclear	Yes	Yes	5/9
Rowan et al. [55]	No	Yes	No	No	Yes	Yes	Yes	Yes	Yes	6/9
Sariakcali et al. [56]	No	Unclear	No	No	Unclear	Yes	Yes	Yes	Unclear	3/9
Scullion et al. [57]	No	No	Yes	Yes	Unclear	Yes	Yes	Yes	Unclear	5/9
Solarz et al. [58]	Unclear	Unclear	No	No	Unclear	Yes	Yes	Yes	Unclear	3/9
Teixeira et al. [59]	No	Yes	No	Yes	Yes	Yes	Yes	Yes	Yes	7/9
Todd et al. [60]	Yes	Unclear	Yes	No	Unclear	Yes	Yes	Yes	Unclear	5/9
Umarov et al. [61]	No	Unclear	No	Yes	Yes	Yes	Yes	Unclear	Yes	5/9
Valtuena et al. [62]	Yes	Unclear	No	No	Unclear	Yes	Yes	Yes	Unclear	4/9
Vitale et al. [63]	Unclear	Unclear	No	Yes	Unclear	Yes	Yes	Yes	Unclear	4/9
Summary score mean										5/9

B
Critical appraisal items
1. Was the sample frame appropriate to address the target population?
2. Were study participants sampled in an appropriate way?
3. Was the sample size adequate?
4. Were the study subjects and the setting described in detail?
5. Was the data analysis conducted with sufficient coverage of the identified sample?
6. Were valid methods used for the identification of the condition?
7. Was the condition measured in a standard, reliable way for all participants?
8. Was there appropriate statistical analysis?
9. Was the response rate adequate, and if not, was the low response rate managed appropriately?

Study	1	2	3	4	5	6	7	8	9	Overall score
Aydin et al. [64]	Yes	No	Yes	No	Unclear	Yes	Yes	Yes	Yes	6/9
Bezuglov et al. [65]	No	Unclear	No	Yes	Unclear	Yes	Yes	Yes	Unclear	4/9
Blume et al. [66]	Yes	No	No	No	Unclear	Yes	Unclear	Yes	Unclear	3/9
Braun et al. [67]	Unclear	No	No	No	Unclear	Yes	Yes	Yes	Unclear	3/9
Brännström et al. [68]	No	No	No	No	Unclear	Yes	Yes	No	Yes	3/9
de Rezende Araújo et al. [69]	No	No	No	Yes	Yes	Yes	Yes	Yes	Yes	6/9
Dubnov-Raz et al. [70]	No	Unclear	No	Yes	Unclear	Yes	Yes	Yes	Yes	5/9
Fields et al. [71]	No	Unclear	No	No	Unclear	Yes	Yes	Yes	Unclear	3/9
Fitzgerald et al. [72]	No	No	No	Yes	Unclear	Yes	Yes	Yes	Unclear	4/9
Geiker et al. [73]	No	Unclear	No	Yes	Unclear	Yes	Yes	Yes	Yes	5/9
Jastrzębska et al. [74]	No	Unclear	No	No	Unclear	No	Yes	Yes	Yes	3/9
Kozlowska et al. [75]	No	Unclear	No	No	Unclear	No	Yes	Yes	Yes	3/9
Seo et al. [76]	No	No	No	No	Unclear	Yes	Yes	Yes	Yes	4/9
Sghaier-Ayadi [77]	Yes	No	No	Yes	Unclear	Yes	Yes	Yes	Unclear	5/9
Wyon et al. [78]	No	No	No	No	Unclear	Yes	Unclear	Yes	Yes	3/9
Summary score mean										4/9

### Prevalence of vitamin D insufficiency and levels of 25(OH)D in adults

**Fig. 2:**
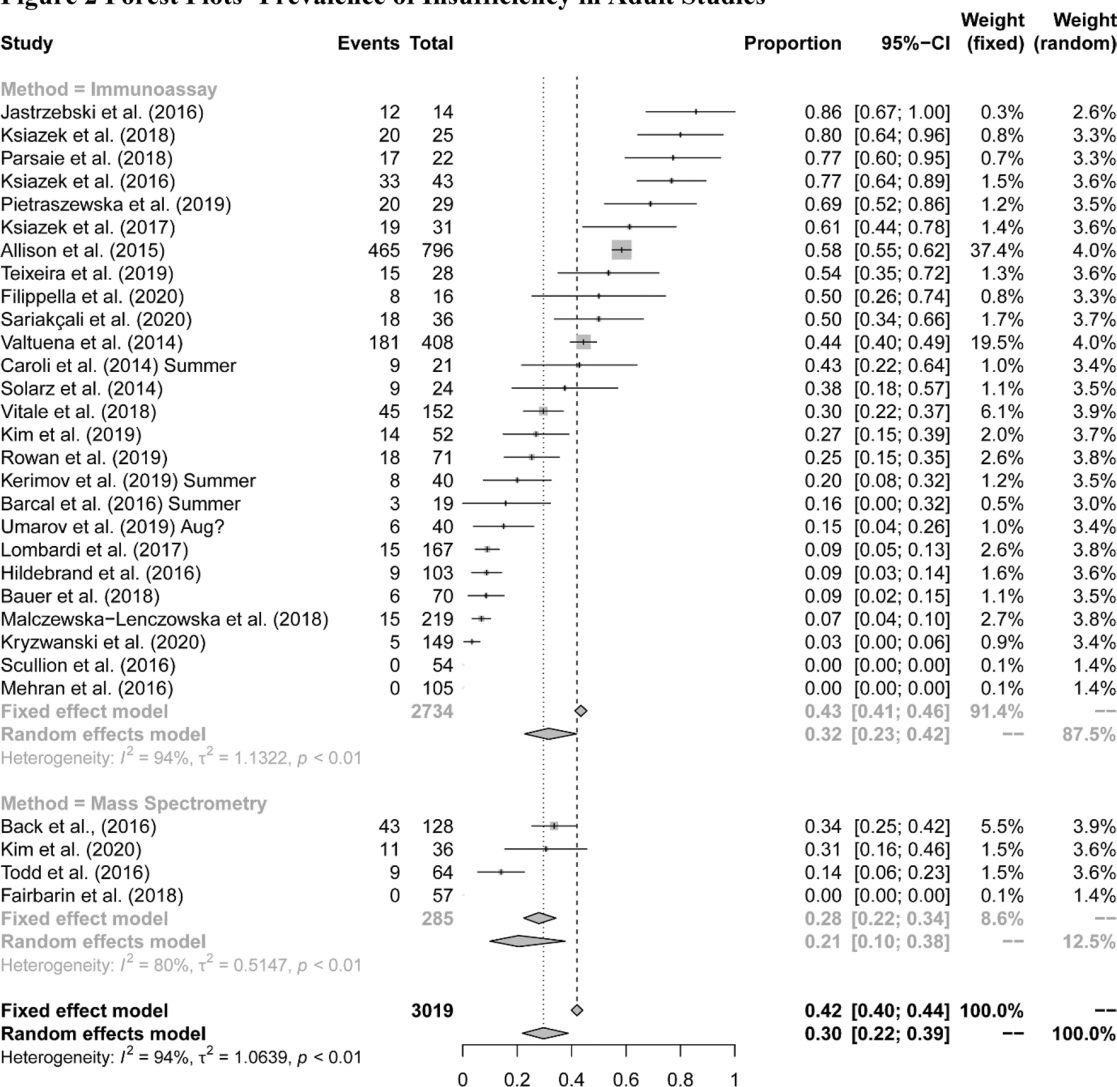
Forest plots—prevalence of insufficiency in adult studies

The pooled mean serum 25(OH)D concentration was 66.4 (SD 30.4) nmol/L. The mean serum 25(OH)D concentration was pooled from 3725 athletes in 27 studies that reported the mean and standard deviation for serum 25(OH)D. The range of means was 33.5 (15.7) to 114.5 (34.3) nmol/L, which shows a large variation in vitamin D concentrations between the studies. (Full details of each adult study provided in Appendix 6.). Using the data from the eligible studies summarized in Table 2, meta-analysis of the prevalence of vitamin D insufficiency within the eligible adult studies was 30% (95% CI 22–39; Fig. 2 using random effects as the *I*^2^ statistic was very high 94%, *p* < 0.001). While there were no differences in prevalence between studies which used immunoassay vs those which used mass spectrometry (*p* = 0.24) it should be remembered that this comparison should be made cautiously since the studies sampled from different populations.

### Prevalence of vitamin D insufficiency and levels of 25(OH)D in adolescents

**Fig. 3:**
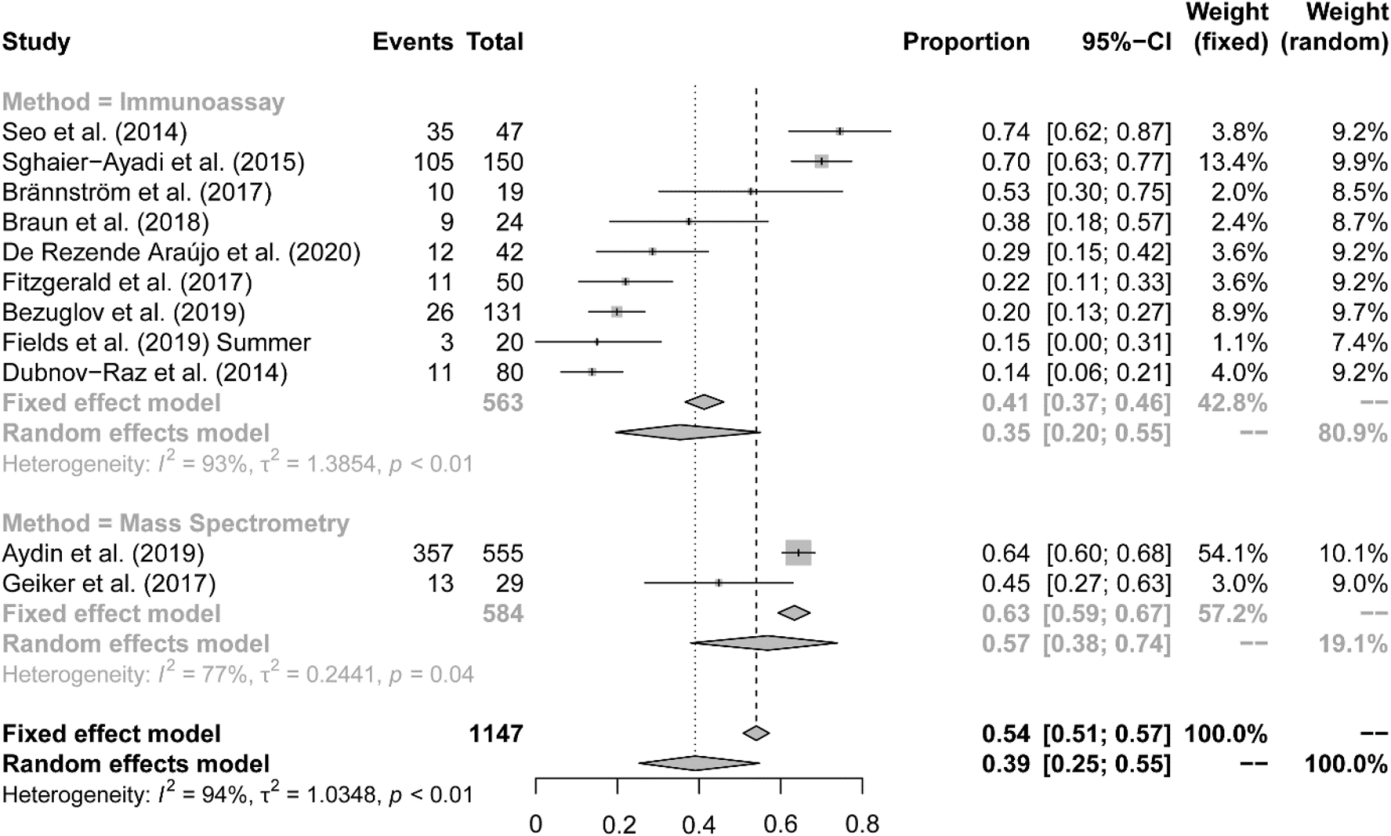
Forest plots**—**prevalence of insufficiency in adolescent studies

The weighted serum 25(OH)D mean (SD) was 60.0 (33.6) nmol/L, calculated from 13 studies that reported the mean (SD) serum 25(OH)D concentration with 1075 participants. The range of means was 41.3 (30.6) to 91.5 (27.8) nmol/L (Full details of each adolescent study provided in Appendix 7). The meta-analysis of the data from the 15 eligible studies summarized in Table 3 provided an estimate of the prevalence of insufficiency among adolescent athletes as 39% (95% CI 25–55%; Fig. 3; the *I*^2^ statistic was 94%, *p* < 0.001 confirming very high heterogeneity).

### Comparisons of adolescents versus adults

The overall prevalence of vitamin D insufficiency was higher among the adolescent than the adult elite athletes, though this difference was not statistically significant. Pooled mean serum 25OH(D) concentrations in adults and adolescents were 66.4 (30.4) nmol/L and 60.0 (33.6) nmol/L, respectively.

### Comparisons of prevalence of vitamin D insufficiency in males versus females

**Fig. 4:**
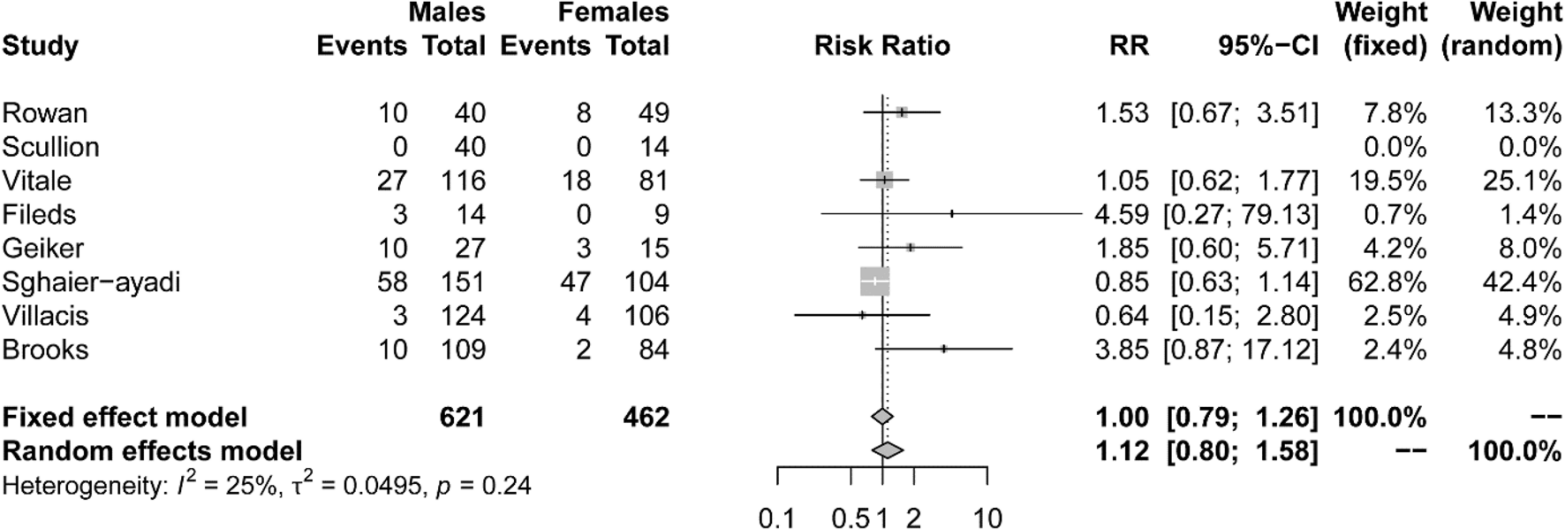
Prevalence of insufficiency differences between males and females within studies

Out of 51 studies (with 5456 athletes), sex/gender was provided in 46 studies. Of these, 24 studies included only male participants, 4 only female participants and 18 studies included both genders, of which only 8 studies provided results separately for males and females and/or could provide such data separately when authors were contacted for this information (Table 5). Sex/gender of study participants was provided for 97% of cases; 69% of participants studied were male (*n* = 3738), and 28% female (*n* = 1529). Not all the studies that included both genders identified the prevalence for genders separately, therefore, both gender and prevalence of insufficiency was known from 2907 males and 682 females. The prevalence of insufficiency for these within-study comparisons for males and females was 34 and 18%, respectively (Table 5.). Meta-analysis of the eight studies summarized in Table 5 (fixed effects, *I*^2^ = 25%) suggested that there was no significant difference in prevalence of insufficiency between males versus females RR (1.00 (95% CI 0.79–1.26), *p* = 0.24 (Fig. 4).

**Table 5:** Differences in prevalence of vitamin D insufficiency between males and females, from within-study differences in the prevalence of insufficiency

Study	Males [(*n* of insufficient males/*n* of all males)]	Females [(*n* of insufficient females/*n* of all females)]	Quality score
Rowan et al.[55]	M: 33% (10/30)	F: 19.5% (8/41)	6/9
Scullion et al. [57]	M: 0% (0/40)	F: 0% (0/14)	5/9
Vitale et al.[63]	M: 30.3% (27/89)	F: 28.6% (18/63)	4/9
Fields et al. [71]	M: 22.7% (3/11	F: 0% (0/9)	3/9
Geiker et al. [73]	M: 59% (10/17)	F: 25% (3/12)	5/9
Sghaier-Ayadi et al. [77]	M: 62.4% (58/93)	F: 82.5% (47/57)	5/9
Brooks et al. [79]	M: 10.1% (10/99)	F: 2.5% (2/82)	6/9
Villacis et al. [80]	M: 2.5% (3/121)	F: 3.9% (4/102)	7/9

## Discussion

### Main study findings and implications

The present review shows that there has been a remarkable increase in the evidence base (51 new eligible studies of elite athletes) in the 7 years since the review of 23 eligible studies by Farrokhyar et al. [11] which searched the literature up to the beginning of 2014. This large new evidence base, which used the more widely accepted and contemporary cut-off to define insufficiency, remains weighted towards males, with a lack of studies of female participants. However, with substantial differences (biological, methodological, sampling, populations) between studies, comparisons of risk of vitamin D insufficiency between groups can only be preliminary at this stage.

Our evidence synthesis suggests that the evidence is of moderately high quality, and the prevalence of vitamin D insufficiency is high, with around one-third of adult elite athletes and more than one-third of adolescent elite athletes with vitamin D insufficiency. These prevalence estimates are probably conservative, since only a relatively small minority of studies recruited participants during winter/spring when insufficiency prevalence will peak [11]*.* Vitamin D has had a high profile in sports science and medicine, in the general population, and in public health policy, in the 7-year period covered by the present review. Despite that increased prominence of vitamin D, the prevalence of insufficiency in these most recent studies was high, though this is not a sports-specific problem. It seems that a relatively high recent profile alone is not sufficient to prevent vitamin D insufficiency in elite sport, and careful implementation of specific strategies to avoid and remedy vitamin D insufficiency, while also avoiding toxicity, are likely to be needed in future [16].

### Comparisons with other evidence

The present systematic review is not directly comparable with the previous systematic review [11] because we used a more contemporary cut-off for serum 25(OH)D of 50 nmol/L to define insufficiency, and had a focus on only the most recent evidence given the dramatically increased profile of vitamin D status in sport, and in public health nutrition policy, in recent years. In addition, the focus on elite athletes only in the present review limits the comparability of the two systematic reviews as Farrokhyar et al. [11] did not define the level of the athletes in the included studies. No previous systematic reviews have considered the potential impact of sex and/or maturity (adolescents vs adults) on risk of vitamin D insufficiency, which also limits comparability with other evidence syntheses. More generally, comparisons between studies are now understood to be more problematic than before because of the apparently substantial differences between laboratories and methods in measurement of serum 25(OH)D, as highlighted recently [10], as well as between-study differences in important factors which influence vitamin D status such as sun exposure. For this reason, we focused our evidence synthesis on prevalence estimates, and our comparisons between the sexes and between adolescents and adults on within-study differences. While we also present pooled summary data for serum 25(OHD) in elite male, female, adult, and adolescent athletes, the between-study variation in analytical methods means that these pooled results should be considered with caution, though meta-analysis of both adult and adolescent studies found that prevalence of insufficiency was similar between the studies which used mass spectrometry and those which used immunoassay. Within-study comparisons of vitamin D status are required for definitive assessment of differences between analytical methods.

### Study and evidence strengths and weaknesses

The present study had a number of strengths. It was a large synthesis which provided robust indications on prevalence of vitamin D insufficiency using a contemporary and relatively widely accepted definition of insufficiency, using only the most recent studies. Second, we considered whether study sample size made any difference to prevalence of insufficiency in adults and adolescents, using an arbitrary cut-off of studies with sample size ≤ 50 or > 50–in the adults the pooled prevalence was much lower in this sensitivity analysis (14 vs 30% overall), but in the adolescents this made little difference (39 vs 36%). Third, we addressed two relatively novel risk factors (sex/gender and maturity). Fourth, we focused on elite athletes and this will increase the generalisability of evidence to elite sport. Finally, we followed good practice in systematic reviewing and evidence appraisal throughout. Funnel plots suggested no significant publication bias for any of the analyses (data not shown).

The present study, and the evidence base, also had a number of weaknesses. First, the evidence was inconclusive on the risk factors for vitamin D insufficiency examined, because of a combination of lack of evidence (e.g., on women), moderate evidence quality, concerns about the measurement and interpretation of serum 25(OH)D measurement and differences between studies (in sampling, latitude, season) mentioned above, and a limited number of studies which included within-study differences between males and females and between adolescents and adults (studies tended to recruit adults or adolescents but not both, and only 5 adult and 3 adolescent studies provided within-study differences between males and females). High risk of bias for prevalence estimation was inevitable for some of the eligible studies because they did not set out with the aim of providing prevalence estimates, but often had other study aims. Second, despite generally wide acceptance of the cut-off of 25(OH)D used in the present study and in the eligible studies, our understanding of how to assess vitamin D status is evolving, and there is some debate as to the value of circulating 25(OH)D concentrations as an indicator of vitamin D status [4]. However, regardless of which cut-off has been used to indicate poor vitamin D status, most previous studies reported large minorities or sometimes majorities of participants having vitamin D status which gave cause for concern [4, 6, 14], as in the general population. While the present review used a much more conservative cut-off than that of the previous systematic review by Farrokhyar et al. [11], the prevalence of insufficiency still gives major cause for concern even with the most recent studies, conducted in an era when vitamin D insufficiency is high on the agenda in both sports nutrition and public health nutrition. In addition, meta-analysis suggested no significant differences in prevalence of insufficiency by broad category of vitamin D status measurement (mass spectrometry vs immunoassay). Third, while the review of Farrokhyar et al. [11] established location/latitude, season, and probably indoor vs outdoor training/competition as risk factors for vitamin D insufficiency, and the present study addressed sex/gender and maturity as potential risk factors, other potential risk factors could not be examined in the present study. The most notable of these is probably skin pigmentation [5], but few of the eligible studies reported the race of the participants, and variation in skin pigmentation may have been one of many factors contributing to the high heterogeneity found by the present review. Some of the studies in the present review reported that participants with darker skin had higher prevalence of insufficiency [39, 50, 54, 71, 80], but future studies will be needed to better understand the relationship between skin pigmentation and vitamin D status. Many other factors are likely to contribute to differences in vitamin D status between studies and in the evidence base available at present these factors have rarely been reported. Differences in status between studies must, therefore, be interpreted cautiously, as they may result from sampling or methodological differences rather than real differences in vitamin D status between samples. Further comparisons within studies will be needed to better understand the factors that mediate and moderate vitamin D status in elite sport, and it would be helpful if future research studies reported a number of relevant factors (e.g., analytical methods; age; gender; maturation status; sun exposure variables such as season, location, training and competition regime predominantly indoor or outdoor; general population vitamin D status, skin pigmentation) so that they can be examined with greater confidence in future meta-analyses. Data sharing would also permit individual participant meta-analyses.

## Conclusions

The present study suggests that at least one-third of elite adult and adolescent athletes have vitamin D insufficiency. These estimates of prevalence are conservative- prevalence will be higher in winter/spring, though lower in summer/fall. The limitations in the evidence on prevalence suggest that there is a need for large surveys of vitamin D status in sport. Such surveys should be sufficiently powered to consider sex/gender and maturity, and should report differences in prevalence by potentially relevant characteristics such as age, skin colour, time of year, sex, and methods of measurement so that risk factors can be understood more confidently. The optimal measure of vitamin D status is still being debated, and a consensus on the optimal measure for use in such future surveys would be informative [10].

## Supplementary Information

Below is the link to the electronic supplementary material.

Supplementary file1 (DOCX 193 KB)
