# Correction to: Prevalence and novel risk factors for vitamin D insufficiency in elite athletes: systematic review and meta-analysis

**DOI:** 10.1007/s00394-022-03021-8

**Published:** 2022-10-11

**Authors:** Tilda Harju, Blair Gray, Alexandra Mavroeidi, Abdulaziz Farooq, John Joseph Reilly

**Affiliations:** 1 University of Strathclyde School of Psychological Sciences & Health, Glasgow, Scotland; 2 FIFA Medical Centre of Excellence, Aspetar, Orthopaedic and Sports Medicine Hospital, Doha, Qatar

## Correction to: European Journal of Nutrition 10.1007/s00394-022-02967-z

The original version of this article unfortunately contained a mistake. Author name Alexandra Mavroeidi was incorrectly written as Alexandra Mavroedi. ORCID id for author Alexandra Mavroeidi should be 0000-0001-5213-1596.

