# Supplementary material for: Prevalence and novel risk factors for vitamin D insufficiency in elite athletes: systematic review and meta-analysis
Source: Eur J Nutr. 2022 Jul 26;61(8):3857–71. doi: 10.1007/s00394-022-02967-z (PMC9596536; doi:10.1007/s00394-022-02967-z)
Supplement: Supplementary file 1 — Supplementary file1 (DOCX 193 KB) [file 394_2022_2967_MOESM1_ESM.docx]

**Appendices/Supplementary Files**

Appendix 1. PRISMA 2009 Checklist

| **Section/topic** | **#** | **Checklist item** | **Reported on page #** |
| --- | --- | --- | --- |
| **TITLE** | | |  |
| Title | 1 | Identify the report as a systematic review, meta-analysis, or both. | 1 |
| **ABSTRACT** | | |  |
| Structured summary | 2 | Provide a structured summary including, as applicable: background; objectives; data sources; study eligibility criteria, participants, and interventions; study appraisal and synthesis methods; results; limitations; conclusions and implications of key findings; systematic review registration number. | 2 |
| **INTRODUCTION** | | |  |
| Rationale | 3 | Describe the rationale for the review in the context of what is already known. | 3-4 |
| Objectives | 4 | Provide an explicit statement of questions being addressed with reference to participants, interventions, comparisons, outcomes, and study design (PICOS). | 5 |
| **METHODS** | | |  |
| Protocol and registration | 5 | Indicate if a review protocol exists, if and where it can be accessed (e.g., Web address), and, if available, provide registration information including registration number. | 5 |
| Eligibility criteria | 6 | Specify study characteristics (e.g., PICOS, length of follow-up) and report characteristics (e.g., years considered, language, publication status) used as criteria for eligibility, giving rationale. | 6 |
| Information sources | 7 | Describe all information sources (e.g., databases with dates of coverage, contact with study authors to identify additional studies) in the search and date last searched. | 6 |
| Search | 8 | Present full electronic search strategy for at least one database, including any limits used, such that it could be repeated. |  |
| 6Study selection | 9 | State the process for selecting studies (i.e., screening, eligibility, included in systematic review, and, if applicable, included in the meta-analysis). | 7 |
| Data collection process | 10 | Describe method of data extraction from reports (e.g., piloted forms, independently, in duplicate) and any processes for obtaining and confirming data from investigators. | 7 |
| Data items | 11 | List and define all variables for which data were sought (e.g., PICOS, funding sources) and any assumptions and simplifications made. | 5-6 |
| Risk of bias in individual studies | 12 | Describe methods used for assessing risk of bias of individual studies (including specification of whether this was done at the study or outcome level), and how this information is to be used in any data synthesis. | 8-9 |
| Summary measures | 13 | State the principal summary measures (e.g., risk ratio, difference in means). | 9-10 |
| Synthesis of results | 14 | Describe the methods of handling data and combining results of studies, if done, including measures of consistency (e.g., I^2^) for each meta-analysis. | 9-10 |

| **Section/topic** | **#** | **Checklist item** | **Reported on page #** |
| --- | --- | --- | --- |
| Risk of bias across studies | 15 | Specify any assessment of risk of bias that may affect the cumulative evidence (e.g., publication bias, selective reporting within studies). | 9,16 |
| Additional analyses | 16 | Describe methods of additional analyses (e.g., sensitivity or subgroup analyses, meta-regression), if done, indicating which were pre-specified. | 9 |
| **RESULTS** | | |  |
| Study selection | 17 | Give numbers of studies screened, assessed for eligibility, and included in the review, with reasons for exclusions at each stage, ideally with a flow diagram. | 9-10,Fig1 |
| Study characteristics | 18 | For each study, present characteristics for which data were extracted (e.g., study size, PICOS, follow-up period) and provide the citations. | 10-11, Tables 2-3 |
| Risk of bias within studies | 19 | Present data on risk of bias of each study and, if available, any outcome level assessment (see item 12). | Table 4 |
| Results of individual studies | 20 | For all outcomes considered (benefits or harms), present, for each study: (a) simple summary data for each intervention group (b) effect estimates and confidence intervals, ideally with a forest plot. | Appendices 6,7 |
| Synthesis of results | 21 | Present results of each meta-analysis done, including confidence intervals and measures of consistency. | Figs 2-4 |
| Risk of bias across studies | 22 | Present results of any assessment of risk of bias across studies (see Item 15) | 16 |
| Additional analysis | 23 | Give results of additional analyses, if done (e.g., sensitivity or subgroup analyses, meta-regression [see Item 16]). | 9,16 |
| **DISCUSSION** | | |  |
| Summary of evidence | 24 | Summarize the main findings including the strength of evidence for each main outcome; consider their relevance to key groups (e.g., healthcare providers, users, and policy makers). | 14 |
| Limitations | 25 | Discuss limitations at study and outcome level (e.g., risk of bias), and at review-level (e.g., incomplete retrieval of identified research, reporting bias). | 15-16 |
| Conclusions | 26 | Provide a general interpretation of the results in the context of other evidence, and implications for future research. | 16-17 |
| **FUNDING** | | |  |
| Funding | 27 | Describe sources of funding for the systematic review and other support (e.g., supply of data); role of funders for the systematic review | 17 |

Appendix 2. PECO Outline

| *Prevalence of Vitamin D insufficiency in Elite level Sport* | | | |
| --- | --- | --- | --- |
| **Population/Setting** | **Exposure** | **Comparator** | **Outcome** |
| Essential/major terms | | | |
| Adult  Elite sportsmen/women | Vitamin D status (deficiency or not) | Deficiency definition | Prevalence |
|  | | | |
|  |  |  |  |
| Related terms | | | |
| Man  Woman  Male  Female  Athletes  Professional  Adolescents | Vitamin D  VITD  Level of Vitamin D  Hypovitaminosis D | Low level  Low status  Insufficiency  25OH(D)  25-hydroxy vitamin D  Deficient | Percentage  Average level  Status  Commonality |

Appendix 3. Inclusion and Exclusion Criteria

| **Inclusion Criteria** | **Exclusion Criteria** |
| --- | --- |
| - Elite-level athletes | - Non-elite athletes or para-athletes |
| - Male and female athletes | - No examination of vitamin D status |
| - Examined vitamin D status   (measured 25(OH)D ) | - Published before January 2014 |
| - English language | - Foreign language |
| - Published after January 2014 | - Animal study |
| - Full text article | - No title, data, abstract, author, or full text available |
| - Human study | - Athletes with health issues e.g. injury, asthma |
| - Peer-reviewed article | - Not peer-reviewed |
|  | - Case reports, review articles, consensus statement, editorial commentary, viewpoints |

Appendix 4. Data Extraction Form

| **Study** | **Sample size** (n),  **Age** [mean(SD)],  **Sport** | **Sex** (%)**, Race:** n | **Country** (latitude**), Season** (month) | **Vitamin D cut-offs** (nmol/L) | **Serum 25(OH)D measurement method** | **Vitamin D status**  [n (%)] | **Serum 25(OH)D level (nmol/L)** [mean (SD)] | **Quality Score** | **Comments** |
| --- | --- | --- | --- | --- | --- | --- | --- | --- | --- |

Appendix 5. Risk of Bias Appraisal Tool

**JBI Critical Appraisal Checklist for Studies Reporting Prevalence Data**


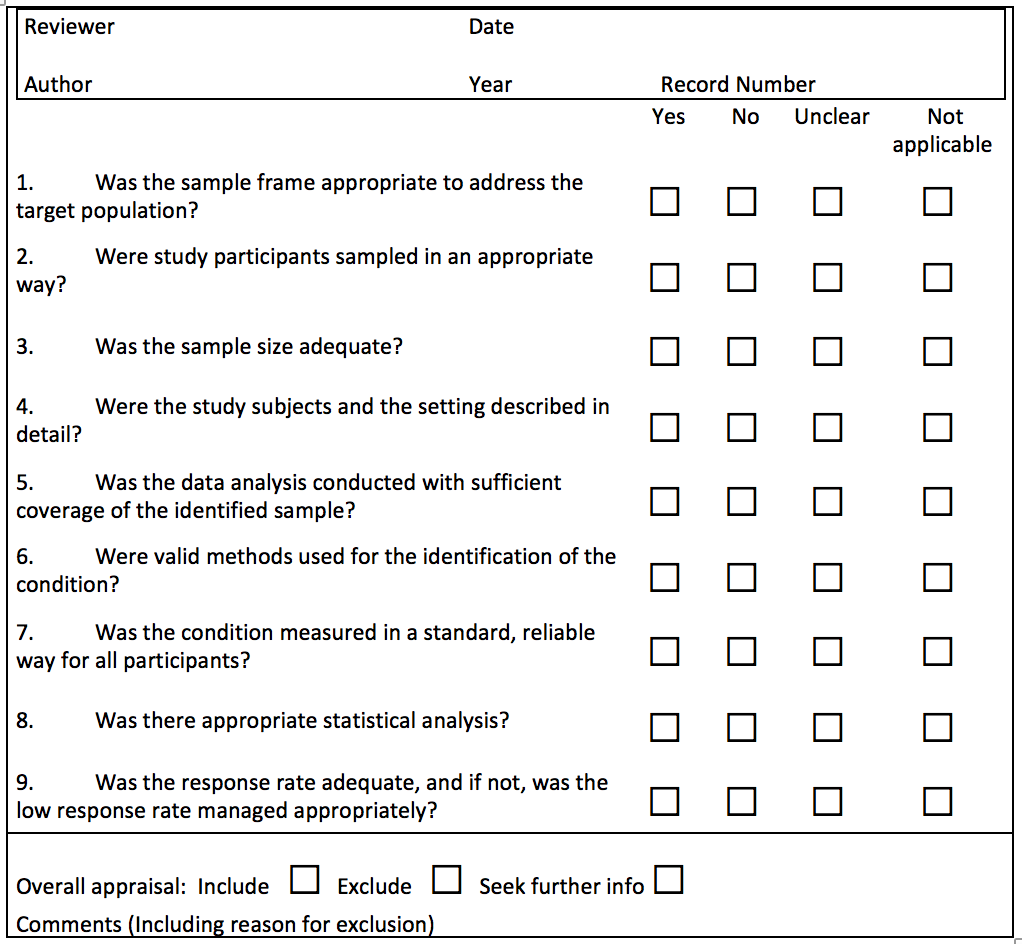


Appendix 6 Overview of eligible adult study characteristics

| **Study** | **Sample size** (n),  **Age [**mean(SD)],  **Sport** | **Sex** (%)**, Race:** n | **Country** (latitude**), Season** (month) | **Vitamin D cut-offs** (nmol/L) | **Serum 25(OH)D measurement method** | **Vitamin D status**  [n (%)] | **Serum 25(OH)D level (nmol/L)** [mean (SD)] | **Quality Score** | **Comments** |
| --- | --- | --- | --- | --- | --- | --- | --- | --- | --- |

| Allison et al. (2015) | 796  24.3 (4.6)  Mixed | Male (100) | Qatar (25^o^N)  Season not given | <25: severely deficient  25-50: deficient  50-75: insufficient  >75: sufficient | Immunoassay | Sev. Def.: 114 (14.3)  Deficient: 351 (44.1)  Insufficient: 206 (25.9)  Sufficient: 125 (15.7) | 50.3 (26.7) | 4/9 | Info received from the author |
| --- | --- | --- | --- | --- | --- | --- | --- | --- | --- |
| Backx et al.,  (2016) | 128  22.0 (3.0)  Mixed | Male (45.3)  Female (54.7)  Light skin: 112  Dark skin: 16 | Netherlands (52°N)  Winter [Mar, Apr] | Deficient: <50  Insufficient: 50–75  Sufficient: >75 | Mass Spectrometry | Deficient: 43 (34)  Insufficient: 46 (36)  Sufficient: 39 (30) | 64.0 (26.0) | 7/9 |  |
| Barcal et al. (2016) | 19  20.9 (2.0) *  Wrestling | Male (100)  Caucasian:15  Spanish Italian: 1  Asian: 2 | USA (43^o^N)  Autumn, Winter, Spring [Sept, Jan, Apr] | Deficient: <50  Insufficient: 50-75  Optimal: >100 | Immunoassay | *September*  Deficient: 2 (11.0)  Insufficient.: 12 (63.0  Sufficient: 5 (26.0)  *January/April*  Deficient: 4 (25)  Insufficient: 11 (69)  Sufficient: 1 (6) | Not given | 7/9 | *From 18 players |
| Bauer et al. (2018) | 70  26.3 (4.9)  Handball | Male (100)  Caucasian: 70 | Germany (50^o^N)  Summer [Jul] | <50:  ≥50-75: insufficient  ≥75-125: sufficient  ≥125: | Immunoassay | <50: 6 (8.6)  Insufficient: 25 (35.7)  Sufficient: 35 (50.0)  ≥125: 4 (5.7) | 83.4 (27.3) | 4/9 |  |
| Caroli et al. (2014) | 21  24.6 (4.3)  Rugby | Male (100)  Caucasian: 20  Hispanic: 1 | Italy (44^o^N)  Summer [Oct],  Winter [Apr] | <25: sev. deficient  ≥25-50: deficient  >50-75: insufficient  >75: sufficient | Immunoassay | *October*  Deficient: 7 (33.3)  Insufficient: 12 (57.1)  Sufficient: 2 (9.5)  *April*  Sev. deficient: 1 (4.8)  Deficient: 11 (52.4)  Insufficient: 9 (42.9) | 47.8 (13.3) | 4/9 |  |
| Fairbairn et al. (2018) | 57  21.2 (2.8)  Rugby | Male (100)  European: 35  N.Z. Māori: 14  Pacific: 8 | New Zealand  (45-46.5°S),  Mar, Apr [Spring] | <25: deficient  <50: insufficient | Mass Spectrometry | Insufficient: 0 | 94.0 (18) | 8/9 |  |

| **Study** | **Sample size** (n)  **Age** [mean(SD)],  **Sport** | **Sex** (%)**, Race:** n | **Country (**latitude**), Season (**month**)** | **Vitamin D cut-offs** (nmol/L) | **Serum 25(OH)D measurement method** | **Vitamin D status**  [n (%)] | **Serum 25(OH)D level (nmol/L)** [mean (SD)] | **Quality Score** | **Comments** |
| --- | --- | --- | --- | --- | --- | --- | --- | --- | --- |

| Filippella et al. (2020) | 16  22.4 (0.7)  Football | Male (100)  Race not given | Italy (42^o^N)  Autumn [Oct],  Spring (May) | <50: deficient  50-75: insufficient | Immunoassay | *October*  Deficient: 4 (25.0)  *May*  Deficient: 12 (75.0) | 79.6 (19.3) | 4/9 |  |
| --- | --- | --- | --- | --- | --- | --- | --- | --- | --- |
| Fishman et al. (2016) | 279  21.5 (1.3)  Basketball | Male (100)  Race not given | USA (38^o^ N)  Spring [May] | <50: deficient  50-80: insufficient  >80: sufficient | Not given | Deficient: 90 (32.3)  Insufficient: 131 (47.0)  Sufficient: 58 (20.8) | 64.0 (25.5) | 2/9 |  |
| Hildebrand et al. (2016) | 103  20.6 (1.9)  Mixed Sports | Male (34)  Female (66)  Caucasian: 78  Af. Amer.: 12  Hispanic: 8  Latin: 3  Asian Pacif.: 2 | USA (35.3-36.2^o^N)  Autumn [Sept-Nov] | <50: deficient  50-75: insufficient  >75: sufficient | Immunoassay | Deficient: 9 (9)  Insufficient: 24 (23)  Adequate: 70 (68) | 90.8 (17.3) | 6/9 |  |
| Jastrzebski  et al. (2016) | 14  23.2 (2.7)  Rowers | Sex not given  Race not given | Poland (52^o^N)  Spring [Mar] | <50: deficient | Immunoassay | Deficient: 12 (85.7) | 33.5 (15.7) | 4/9 |  |
| Kerimov et al. (2019) | 40  22.1 (0.3)  Wrestling | Male (100)  Race not given | Uzbekistan (41^o^N)  Summer [Aug],  Winter [Jan] | ≤50: deficient  50-75: insufficient  ≥75-375: sufficient  >375: intoxication | Immunoassay | *August*  Deficient: 4 (10.0)  Insufficient: 32 (80.0)  Sufficient: 4 (10.0)  *January*  Deficient: 12 (30.0)  Insufficient: 28 (70.0) | Not given | 5/9 |  |
| Kim et al. (2019) | 52  23.8 (2.8)  Volleyball | Male (100)  Race not given | South Korea (36^o^N)  Season not given | <50: deficient  50-75: insufficient  >75: sufficient | Immunoassay | Deficient: 14 (26.9)  Insufficient: 24 (46.2)  Sufficient: 14 (26.9) | 63.0 (20.8) | 3/9 |  |
| Kim et al. (2020) | 36  22.6 (3.0)  Basketball | Male (100)  Race not given | South Korea (36^o^N)  Spring [Apr] | <50: deficient  50-80: insufficient  >80: sufficient | Mass Spectrometry | Deficient: 11 (30.5)  Insufficient: 15 (41.7)  Sufficient: 10 (27.8) | 61.8 (18.0) | 3/9 |  |
| Kryzwanski et al. (2020) | 149  25.5 (0.6)  Track and Field | Male (53)  Female (47)  Caucasian: 149 | Poland (52^o^N),  South Africa (27^o^S),  Spain (28^o^N)  All Seasons | <50: deficient  50-75: insufficient  75-125: normal  >250: toxic | Immunoassay | Deficient: 5 (3.2)  Insufficient: 49 (32.9) | 90.0 (31.0) | 6/9 |  |
| **Study** | **Sample size** (n),  **Age** [mean (SD)], **Sport** | **Sex** (%),  **Race:** n | **Country (**latitude**), Season (**month**)** | **Vitamin D cut-offs** (nmol/L) | **Serum 25(OH)D measurement method** | **Vitamin D status**  [n (%)] | **Serum 25(OH)D level (nmol/L)** [mean (SD)] | **Quality Score** | **Comments** |
| Ksiazek et al. (2016) | 43  22.7 (5.3)  Football | Male (100)  Race not given | Poland (51^o^N)  Winter [no month] | <50: deficient  50-75: insufficient  75-125: physiology. norm | Immunoassay | Deficient: 33 (76.7)  >50: 10 (23.3) | 42.3 (21.0) | 3/9 |  |
| Ksiazek et al. (2017) | 31  26.3 (5.9)  Football | Male (100)  Race not given | Poland (51^o^N)  Winter [no month] | <50: deficient  50-75: insufficient  75-150: normal | Immunoassay | Deficient: 19 (61.3)  >50: 12 | 52.8 (21.5) | 2/9 |  |
| Ksiazek et al. (2018) | 25  21.9 (9.8)  Judo | Sex not given  Race not given | Poland (51^o^N)  Winter [no month] | <50: deficient  50-75: insufficient  75-125: physiology. norm | Immunoassay | Deficient: 20 (80.0) | 43.5 (13.0) | 3/9 |  |
| Lombardi et  al. (2017) | 167  25.1 (4.7)  Football | Male (100)  Race not given | Italy (42^o^N)  All Seasons [Jan-Apr, July, Aug, Oct-Dec] | <50: deficient  50-75: insufficient | Immunoassay | Deficient: 15 (8.9)  Insufficient: 55 (32.9) | Not given | 3/9 |  |
| Malczewska-Lenczowska  et al. (2018) | 219  20.0 (4.4)  Mixed Sports | Female (100)  Caucasian: 219 | Poland (52^o^N)  All Seasons [Jan-Dec] | <25: deficient  25-75: insufficient | Immunoassay | Deficient: 4 (1.8)  25-50: 11 (5.0) *  50-75: 104 (47.5)  >75: 100 (4) | 74.8 (23.8) | 7/9 | *Info received from the author |
| Maroon et al. (2015) | 80  26.5 (3.7)  Am. Football | Male (100)  Black: 67  White: 13 | USA (37^o^N)  Winter, Summer, Autumn [Feb-Sept] | <50: deficient  50-80: insufficient  >80: sufficient | Not given | Deficient: 21 (26.3)  Insufficient: 34 (42.5)  Sufficient: 25 (31.3) | 68.5 (29.3) | 3/9 |  |
| Mehran et al. (2016) | 105  25.5 (4.4)  Hockey | Male (100)  White: 101 | Canada/USA (56^o^N/37^o^N  Summer [Sept] | <50: deficient  50-80: insufficient  >80: sufficient  >100: ideal | Immunoassay | Deficient: 0  Insufficient: 14 (13.3)  Sufficient: 91 (86.7)  Ideal: 68 (64.8) | 114.5 (34.3) | 4/9 |  |
| Parsaie et al. (2018) | 22  27 (4.5)*  Football | Sex not given  Race not given | Iran (32^o^N)  Winter [no month] | <50: deficient  50-75: insufficient  >75: sufficient | Immunoassay | Deficient: 17 (77.3)  Insufficient: 5 (22.7) | 26.7 (no SD) | 4/9 | *Median (IRQ) |
| Pietraszewska et al. (2019) | 29  26.6 (5.8)  Football | Male (100)  Race not given | Poland (52° N)  Winter [no month] | <50: deficient  50-75: insufficient  >75: normal values | Immunoassay | Deficient: 20 (69.0)  Insufficient: 9 (31.0) | 45.8 (11.0) | 3/9 |  |
| Rebolledo et al. (2018) | 214  22.1 (1.0)  Am. Football | Male (100)  Af. American: 167  White/Polyn: 47 | USA (38° N)  Season not given | <50: deficient  50-80: insufficient  ≥80: normal values | Not given | Deficient: 22 (10.2)  Insufficient: 104 (48.5) | 79.5 (30.8) | 5/9 |  |

| **Study** | **Sample size (n), Age [mean (SD)], Sport** | **Sex (%), Race: n** | **Country (latitude), Season (month)** | **Vitamin D cut-offs** (nmol/L) | **Serum 25(OH)D measurement method** | **Vitamin D status**  [n (%)] | **Serum 25(OH)D level (nmol/L)** [mean (SD)] | **Quality Score** | **Comments** |
| --- | --- | --- | --- | --- | --- | --- | --- | --- | --- |

| Rowan et al. (2019) | 71  31.1 *  Ballet Dancers | Males (42)  Females (58)  Race not given | UK (55° N)  Winter [no month] | <50: deficient  50-70: insufficient  >70: normal values | Immunoassay | Deficient: 18 (25.4)  Insufficient: 15 (21.1)  Normal: 38 (53.5) | 76.7 (27.3) | 6/9 | *no SD given |
| --- | --- | --- | --- | --- | --- | --- | --- | --- | --- |
| Sariakçali  et al. (2020) | 36  23.3 (3.5)  Football | Male (100)  Race not given | Turkey (40° N)  Summer [no month] | <50: deficient  50-75: insufficient  >75: sufficient | Immunoassay | Deficient: 18 (50)  Insufficient: 14 (38.9)  Sufficient: 4 (11.1) | 52.0 (15.8) | 3/9 |  |
| Scullion et al. (2016) | 54  22.9 (3.2)  Rowing, Rugby | Male (75)  Female (25)  Fair Skin: 14  Medium: 29  Dark: 10 | New Zealand (41° 2)  Summer [Jan] | <50: deficient  >75: beneficial | Immunoassay | Deficient: 0 | 100.4 (20.4) | 5/9 | no mention about gender  *53 |
| Solarz et al. (2014) | 24  26.5 (3.4)  Football | Male (100)  Race not given | Poland (52° N)  Winter [Apr] | <50: deficient  50–75: insufficient  75–150: normal | Immunoassay | Deficient: 9 (37.5)  Insufficient: 11 (45.8)  Normal: 4 (16.7) | 62.6 (24.8) | 3/9 |  |
| Teixeira et al. (2019) | 28  24 (5.75)*  Football | Male (100)  Med./Olive: 21  Dark: 7 | Portugal (40°N)  Winter [Dec] | <50: deficient  50:75: inadequate  75-250: sufficient | Immunoassay | Deficient: 15 (53.6)  Inadequate:10 (35.7)  Sufficient: 3 (10.7) | Not given | 7/9 | *Median & (IQR) |
| Todd et al. (2016) | 64  25.0 (5.0)  Mixed* | Sex not given  Race not given | Ireland (53° N)  Year round [Feb-May, Sept, Nov] | <30: deficient  30-50: insufficient  >50: sufficient | Mass Spectrometry | Deficient: 1 (2)  Insufficient: 8 (12)  Sufficient: 55 (86) | 76.5 (27.0)** | 5/9 | *Cricket, Boxing, Rugby  **from 92 blood samples |
| Umarov et al. (2019) | 40  20.7 (1.3)  Synchronized Swimming,  Swimming | Female (100)  White: 26  Black: 14 | Uzbekistan (41° N)  Winter, Summer  [no month] | ≤50: deficient  50-75: insufficient  ≥75-425: sufficient | Immunoassay | *August*  Deficient 2 (5.0)  Insufficient 36 (90.0)  Sufficient 2 (5.0)  *February*  Deficient 10 (25.0)  Insufficient: 30 (75.0) | Not given | 5/9 |  |
| Valtuena et al. (2014) | 408  22.8 (8.4)  Mixed | Male (58)  Female (42)  Race not given | Spain (41°N)  All Seasons [no month] | <27.5: sev. deficient  <50: mod. deficient  50-75: insufficient  >75: optimal | Immunoassay | Sev. deficient.: 23 (5.6)  Mod. Def.: 158 (38.7)  Insufficient.: 154 (37.7)  Optimal: 73 (17.9) | 56.7(23.5) | 4/9 |  |
| Vitale et al. (2018) | 152  24.1 (3.2)  Alpine Skiing | Male (59)  Female (41)  Caucasian: 152 | Italy (42° N)  All Seasons  [Jan-Jul, Sept-Dec] | <50: deficient  50-75: insufficient | Immunoassay | Deficient: 45 (29.6) *  Insufficient: 77 (50.7) | 67.8 (19.8) | 4/9 | *Athletes had serum 25(OH)D levels  <50 nmol/L at least once during the study period |

Unweighted data:1167/3592 study participants had insufficiency.

Appendix 7 Overview of eligible adolescent study characteristics

| **Study** | **Sample size** (n),  **Age [**mean(SD)],  **Sport** | **Sex** (%)**, Race:** n | **Country** (latitude**), Season** (month) | **Vitamin D cut-offs** (nmol/L) | **Serum 25(OH)D measurement method** | **Vitamin D status**  [n (%)] | **Serum 25(OH)D level (nmol/L)** [mean (SD)] | **Quality Score** | **Comments** |
| --- | --- | --- | --- | --- | --- | --- | --- | --- | --- |

| Aydin et al. (2019) | 555  15.9*  Mixed | Male (41)  Female (59)*  Race not given | Turkey (41^o^N)  Autumn, Winter  [Sept-Feb] | | <25: severely deficient  25-50: deficient  50-75: insufficient  >75: sufficient | Mass Spectrometry | | Sev. Def.: 120 (21.6)  Deficient: 237 (42.7)  Insufficient: 108 (19.5)  Sufficient: 90 (16.2) | 53.5 (35.8)** | | 6/9 | | *age and genders from all athletes  ** from 5-17 age group | |  |
| --- | --- | --- | --- | --- | --- | --- | --- | --- | --- | --- | --- | --- | --- | --- | --- |
| Bezuglov et al. (2019) | 131  15.6 (2.4)  Football | Male (100)  White: 131 | | Russia (55°N)  Winter [Dec] | <50: deficient  50-75: insufficient  75-150: normal  >150: excess | Immunoassay | Deficient: 26 (19.9)  Insufficient: 30 (22.9)  Normal: 35 (26.7)  Excess: 40 (30.5) | | | Not given | | 4/9 | |  | |
| Blume et al. (2019) | 146  14.7 (1.7)  Mixed | Male (66)  Female (34)  Race not given | | Germany (51^o^N)  All Seasons  [no month] | <50: deficient | Not given | Deficient: 12 (8.2) | | | 91.5 (27.8) | | 3/9 | | Results from 356 visits | |
| Braun et al. (2018) | 24  14.8 (0.7) *  Football | Female (100)  Race not given | | Germany (51^o^N)  All seasons  [no month] | <12: severely deficient  12-30: deficient  30-50: inadequate  >50: adequate | Immunoassay | Inadequate: 9 (37.5)  Adequate: 15 (62.5) | | | 50.2 (16.6) | | 3/9 | | *Mean age from 56 participant | |
| Brännström et al. (2017) | 19  15.3 (0.7)  Football | Female: 100  Race not given | | Sweden (63°N)  Winter [Jan-Mar] | Deficient: <25  Insufficient: 25–75  Sufficient: 75–250 | Immunoassay | <50: 10 *  50-75: 7  Sufficient: 1 | | | 50.5 (12.8) | | 3/9 | | *info received from author | |
| De Rezende Araújo et al. (2020) | 42  17 (5.3)  Dance | Male (11.9)  Female (88.1)  Caucasian: 40  Other: 2 | | Brazil (16^o^S)  Autumn [Oct] | <25: deficient  25-75: insufficient  >75: normal | Immunoassay | <50: 12 (28.6)*  Insufficient: 4 (9.5)  Normal: 26 (61.9) | | | 82.1 (40.5) | | 6/9 | | *info received from the author | |
| Dubnov-Raz et al. (2014) | 80  14 (1.6)  Swimming | Male (65)  Female (35)  Race not given | | Israel (32°N,  31°N)  Autumn [Oct] | <50: deficient  50-75: insufficient  >75: sufficient | Immunoassay | Deficient: 11 (14.0)  Insufficient: 42 (53.0)  Sufficient: 27 (34.0) | | | 69.3 (17.8) | | 5/9 | |  | |
| Fields et al. (2019) | 20  19.9 (1.2)  Basketball | Male (55)  Female (45)  Race not given | | USA (38^o^N)  Summer [Jul],  Autumn [Oct] | ≤50: deficient  50-75: insufficient  ≥75: sufficient | Immunoassay | *July*  Deficient: 1 (5.0)  Insufficient:11 (55.0)  Sufficient: 8 (40.0)  *October*  Deficient: 4 (20.0)  Insufficient: 10 (50.0)  Sufficient: 6 (30.0) | | | 77.3 (35.1) | | 3/9 | |  | |

| **Study** | **Sample size** (n),  **Age [**mean(SD)],  **Sport** | **Sex** (%)**, Race:** n | **Country** (latitude**), Season** (month) | **Vitamin D cut-offs** (nmol/L) | **Serum 25(OH)D measurement method** | **Vitamin D status**  [n (%)] | **Serum 25(OH)D level (nmol/L)** [mean (SD)] | **Quality Score** | **Comments** |
| --- | --- | --- | --- | --- | --- | --- | --- | --- | --- |

| Fitzgerald  et al. (2017) | 50  17.2 (0.9)  Ice Hockey | Male (100)  Caucasian: 50 | Poland (50^o^N)  Autumn [Oct] | <50: deficient  50-75: insufficient  >75: sufficient: | Immunoassay | Deficient: 11 (22.0)  Insufficient: 20 (40.0)  Sufficient: 19 (38) | 75.8 (37.3) | 4/9 |  |
| --- | --- | --- | --- | --- | --- | --- | --- | --- | --- |
| Geiker et al. (2017) | 29  18.6 (2.0)  Swimming | Male (59)  Female (41)  Caucasian: 29 | Denmark (55^o^N)  Spring [Mar, Apr] | <50: insufficient  >50: sufficient | Mass Spectrometry | Insufficient: 13 (45.0)  Sufficient: 16 (55.0) | 52.6 (18.3) | 5/9 |  |
| Jastrzebska et al. (2016) | 36  17.5 (0.6)  Football | Sex and race not given | Poland (52^o^N)  Winter [Month not given] | Not classified | Immunoassay | <50 nmol/L: 22 (61.1)  >50 nmol/L: 14 (38.9) | 48.1 (12.4) | 3/9 |  |
| Kozłowska et al. (2019) | 36  17.8 (3.9)  Tennis | Male (100)  Race not given | Poland (52^o^N)  Winter, Spring, Autumn [Jan, May, Sept] | Not classified | Mass Spectrometry | <50 nmol/L: 22 (61.1)  >50 nmol/L: 14 (38.9) | 41.3(30.6)* | 3/9 | *from 23 players <18 years old |
| Seo et al. (2014) | 47  16.7 (0.8)  Taekwondo | Male: 100  Race not given | Republic of Korea (37°N)  Winter (Nov) | <30: deficient  30-50: insufficient  >50: adequate | Immunoassay | Deficient: 5 (10.6)  Insufficient: 30 (63.8)  Adequate: 12 (25.5) | 43.8 (13.3) | 4/9 |  |
| Sghaier-Ayadi et al. (2015) | 150  18.0 (2.0)  Mixed | Male (62)  Female (38)  Fair Skin: 75  Brown Skin: 71  Dark Skin: 4 | Tunisia (33^o^N, 37^o^N)  Winter [Jan, Feb] | <25: sev. deficient  <50: deficient  <75: inadequate  >75: not given | Immunoassay | Sev. deficient: 22 (14.7)  Deficient: 83 (55.3)  Inadequate: 32 (21.3)  >75: 13 (8.7) | 43.3 (20.1) | 5/9 |  |
| Wyon et al. (2018) | 74  17.0 (0.9)  Dance | Male (57)  Female (43)  Race not given | England (52^o^N)  Winter, Spring  [Jan, Apr] | <25: deficient  25-75: insufficient  >75: sufficient | Not given | Deficient: 4 (5.4)  Insufficient: 52 (70.3)  Sufficient: 18 (24.3) | Not given | 3/9 |  |

Unweighted data: 652/1432 study participants had insufficiency.
